# Experimentally advancing morning emergence time does not increase extra-pair siring success in blue tit males

**DOI:** 10.1093/beheco/arad006

**Published:** 2023-02-27

**Authors:** Peter Santema, Bart Kempenaers

**Affiliations:** Max Planck Institute for Ornithology, Department of Behavioural Ecology and Evolutionary Genetics, Seewiesen, Germany; Edward Grey Institute of Field Ornithology, University of Oxford, Oxford, UK; Max Planck Institute for Ornithology, Department of Behavioural Ecology and Evolutionary Genetics, Seewiesen, Germany

**Keywords:** artificial light at night, *Cyanistes caeruleus*, extra-pair paternity, mixed reproductive strategy, morning activity, sexual selection

## Abstract

Extra-pair paternity occurs frequently in socially monogamous birds, but there is substantial variation in extra-pair siring success among males. Several studies have shown that siring success relates to the timing of morning activity, with the earliest active males being more successful, suggesting that early activity is important for acquiring extra-pair copulations. However, these studies are correlational, and it, therefore, remains unclear whether the relationship between timing and extra-pair siring success is causal. An alternative explanation is that successful extra-pair sires tend to be active earlier (e.g., because they are of high quality or in good condition), but that early activity in itself does not increase siring success. We experimentally advanced the emergence time of male blue tits by exposing them to light about half an hour before their natural emergence time. Although males that were exposed to the light treatment emerged from their roost substantially earlier than males that were exposed to a control treatment, light-treated males were not more likely to sire extra-pair offspring. Furthermore, whereas control males showed the expected relation between emergence time and siring success (although not statistically significant), there was no relation between emergence time and extra-pair siring success among light-treated males. Our results suggest that the timing of emergence from the roost is not an important factor underlying extra-pair siring success.

## Teaser

Offspring sired outside the pair-bond occur regularly in socially monogamous birds. Males that are active earlier in the day tend to be more successful in siring extra-pair young, but it is unclear whether this relationship is causal. We experimentally advanced the emergence time of male blue tits, but this did not increase their extra-pair siring success. Our results challenge the idea that early activity per se increases extra-pair siring success

## INTRODUCTION

Extra-pair offsprings, which result from copulations outside the pair bond, are common among socially monogamous birds (Griffith et al. 2002; Brouwer and Griffith 2019; Lifjeld et al. 2019; Valcu et al. 2021). By siring offspring with one or more females other than their own social mate, males can increase their reproductive output at the expense of other males. Within a population, males vary substantially in extra-pair siring success, and extra-pair paternity can thus influence the variance in reproductive success between males (Birkhead and Møller 1998), and hence the intensity of sexual selection (Webster et al. 1995). The occurrence of extra-pair paternity has, therefore, attracted much interest and a large body of research has aimed to understand its evolutionary causes and consequences (Petrie and Kempenaers 1998; Westneat and Stewart 2003; Brouwer and Griffith 2019; Lifjeld et al. 2019). To understand the consequences of extra-pair paternity for sexual selection, we need to understand why some males are more successful at siring extra-pair offspring than others (Webster et al. 1995; Schlicht and Kempenaers 2013).

Several studies have suggested that extra-pair siring success is related to the timing of morning activity (Poesel et al. 2006; Dolan et al. 2007; Kempenaers et al. 2010; Schlicht et al. in press). For instance, blue tit males that had an earlier onset of singing during the peak fertile period were more likely to sire extra-pair offspring and sired offspring with more females (Poesel et al. 2006). A recent study (Schlicht et al. in press) showed that earlier emergence from the roost is also associated with higher extra-pair siring success and that this effect is independent of male age, a factor associated with both earlier activity and higher extra-pair siring success (e.g., Steinmeyer et al. 2010; Cleasby and Nakagawa 2012). Moreover, a study on the influence of artificial street lighting reported that blue tit males that were exposed to light started singing earlier, were more likely to sire extra-pair young, and had more extra-pair mates than those not exposed to artificial light (Kempenaers et al. 2010). Studies on great tits *Parus major*, blackbirds *Turdus merula,* and willow tits *Parus montanus*, showed that males started singing substantially earlier in the period just before females started egg-laying, also suggesting that there is a link between the timing of activity and mating behavior (Cuthill and McDonald 1990; Welling et al. 1995; Halfwerk et al. 2011). The notion that the early morning is important for extra-pair copulations is further corroborated by observations that extra-pair copulations often occur around dawn (Smith 1988; Double and Cockburn 2000; Mennill et al. 2004).

Although previous work suggests that the timing of activity is important, the correlation between extra-pair siring success and morning activity does not imply causality. Extra-pair siring success and early morning activity may both be related to a third factor, for instance, body condition or other indices of quality. Thus, the timing of the activity by itself might have no influence on extra-pair siring success. Previous work on blackbirds and black-capped chickadees, *Poecile atricapillus*, showed that males that were supplementary fed started singing earlier in the morning (Cuthill and Macdonald 1990; Grava et al. 2009), suggesting that early activity may indeed be a consequence of being in better condition. A study on Eastern kingbirds, *Tyrannus tyrannus*, found that males that sang the earliest also had the largest tarsi and longest flight feathers (Murphy et al. 2008), suggesting a link between singing time and dominance or competitiveness. Another indication that the relation between male emergence time and extra-pair success may not be causal is that females typically emerge from their roost much later than males (Mace 1987; Steinmeyer et al. 2010; Schlicht et al. 2014a; Stuber et al. 2015; Hau et al. 2017), and it remains, therefore, unclear how emerging earlier would give males an advantage in accessing potential extra-pair mates (Mace 1987; Greives et al. 2015). Thus, although several studies reported a link between early activity and extra-pair siring success, the hypothesis that early activity per se improves extra-pair siring success remains to be tested experimentally.

The aim of this study is to experimentally test whether earlier emergence leads to higher extra-pair siring success in blue tits. Blue tits are socially monogamous, but extra-pair paternity is common (Kempenaers et al. 1992; Delhey et al. 2003; Schlicht et al. 2014a). Each year, about a third of the breeding males sire at least one extra-pair young (Schlicht and Kempenaers 2013), and the timing of emergence from the roost is correlated with extra-pair siring success, independently of male age (Schlicht et al. in press). We advanced the timing of emergence from the roost by exposing males roosting in a nest-box to artificial light about half an hour before their natural emergence time. Using an automated monitoring system, we obtained precise information on the emergence time of each experimental and control male. We then compared extra-pair siring success of males that had been exposed to the experimental treatment and those that had been exposed to a control treatment by genotyping all nestlings and adults in the population.

## METHODS

### Study system

The study took place in 2021 in Westerholz, an oak-dominated forest in Southern Germany near Landsberg am Lech (48° 08’ 26’’ N 10° 53’ 29’’ E) where we have studied the breeding behavior of blue tits since 2007. The study site contained 276 nest-boxes of which 143 were occupied (i.e., had a nest with at least one egg). For the purpose of the experiment, we rearranged nest-boxes in pairs such that there were always two boxes on the same tree facing in opposite directions. We thus provided a potential roosting box for males near the breeding box, that is, near the typical roost site of the female. This setup was chosen to increase the probability that a male would roost in a nest-box, which is necessary to be able to conduct the experiment. Throughout the breeding season, we checked nest-boxes regularly to monitor the onset and progress of nest building, the date of the first egg, clutch size, the date of first hatching, and breeding success. The social parents for each nest were determined based on nest visit data and confirmed with parentage analysis. For a more detailed description of the study site and general field procedures, see Schlicht et al. (2012).

### Emergence time monitoring system

All nest-boxes were equipped with an automated monitoring system, consisting of a radio frequency identification reader, a clock, light barriers, and a data storage device (Loës et al. 2019). All males included in the study were equipped with a passive integrated transponder (SMARTRAC glass tag 134 kHz, EM4305, 8.3 × 1.41 mm, 0.03 g) which was inserted on the back under the skin (Schlicht and Kempenaers 2015). Birds had been caught either during previous breeding attempts when provisioning offspring, or prior to the breeding season using mist-nets and snap-traps, or when roosting in a nest-box. Age at capture (yearling or older) was determined based on plumage characteristics (Cramp and Perrins 1993). Whenever a tagged bird passed through the nest hole, its identity and the associated time and date were automatically stored (see Schlicht et al. 2012 for details). This provided uniquely comprehensive data on roosting behavior and morning emergence times for individuals roosting in the nest-boxes. To identify nest-boxes that were used for roosting by a male, we extracted the data once a week.

### Emergence time manipulation

**Figure 1:**
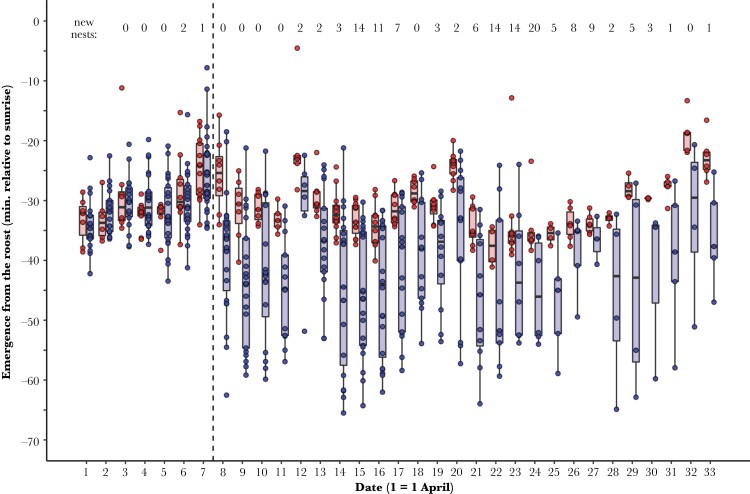
Emergence times of males that were exposed to the experimental light treatment (blue, *N* = 31) and males that were exposed to the control treatment (red, *N* = 15). The vertical dashed line indicates the start of the light treatment. The numbers at the top show for each day the number of females that started a clutch. Boxplots show median, 1st and 3rd quartile and 1.5 times the inter-quartile range. Dots show the raw data.

We advanced the emergence time of experimental males by exposing them to light early in the morning (Schlicht et al. 2014a). Most extra-pair fertilizations occur shortly before the onset of egg laying or early in the laying order (Magrath et al. 2009; Schlicht et al. 2014a), and we, therefore, advanced the emergence time of males during the period shortly before females started new clutches (see Figure 1). A warm-white diffuse LED (Nichia NSPL515DS, Nichia Corporation, Tokushima, Japan; typical luminous intensity: 1.9 cd) was attached to the lid inside the nest-box. The LED was connected via a cable to a timer (relay: EATON EASY512-DA-RC, Moeller GmbH, Bonn, Germany) and a battery, both of which were placed in a plastic box on the ground below the nest-box. The device was programmed to switch on approximately an hour before sunrise (range: 1 h and 10 min to 1 h before sunrise). At least once per week, we adjusted the timer to advance in the time of sunrise over the course of the experiment and to replace the batteries. Nest-boxes of control males were equipped with a non-functional LED that was not connected to a timer or battery and had a shorter cable and no box on the ground.

We started the experiment on 8 April (2 days after the first female in the population had started laying) and continued until 3 May (when all females had commenced egg laying and most had finished laying). We did not continue the treatment until all females had finished egg laying, because in blue tits extra-pair paternity occurs almost exclusively among the first-laid eggs (Magrath et al. 2009; Santema et al. 2021). A priori we decided to assign two-thirds of all males that were identified to be roosting in a nest-box to the experimental treatment and one-third to the control treatment. Not all males roosted in the nest-box each night of the experimental period, and males that were identified as roosting in a nest-box after the onset of the experiment were still included. Males roosted on average 10 nights in the nest-box (range 1–26) during the experimental period. In total, we manipulated 46 males (31 experimental, 15 control) for a total of 451 mornings (276 light-treated, 175 control).

### Paternity analysis

We took a blood sample (ca. 5–10 µL) from all adults when they were caught for the first time and from all nestlings when they were ringed and weighed at 14 days of age. We also collected dead nestlings and unhatched eggs and genotyped them if sufficient DNA could be extracted. To assess parentage, we compared the genotypes from parents and their putative offspring using a set of 14 microsatellite markers (see Schlicht et al. in press). Of the 46 males that were included in the experiment, 15 sired extra-pair offspring. Twelve of these males sired extra-pair offspring with only one female, while the remaining three sired offspring with two different females. Because only few males sired extra-pair offspring with multiple females, and because the number of young sired with a particular female is partly determined by post-copulatory processes (sperm competition), we only examined whether or not a male had sired at least one extra-pair young (yes/no). We also genotyped all breeding males that did not roost in a nest-box (*n* = 92)—and were thus not part of the emergence time experiment—and used these as an additional control group.

### Statistical analyses

All statistical analyses were performed with the software R (version 4.1.0; R Development Core Team 2021), using the lme4 package (Bates et al. 2015).

First, we examined whether the experimental treatment affected the time males emerged from the roosting box, as intended. We constructed a linear mixed model with emergence time (in minutes relative to sunrise) as the response variable, treatment (light or control) as a fixed effect, and male identity as a random intercept. To confirm that there was no difference in emergence time between the two groups before the onset of the experiment, we performed the same analyses using emergence time data from 7 days before the start of the treatment.

Second, we examined whether the light treatment affected the probability of siring extra-pair young. We constructed a general linear model with a binomial error structure, with whether a male sired at least one extra-pair young (yes/no) as the response variable and with treatment (light, control, or untreated) as the fixed effect. Because male age is a known predictor of extra-pair siring success, with yearlings being much less likely to sire extra-pair young than adults, we also included male age (yearling or adult) as a fixed effect. Only few yearling males were included in this study (control: *n* = 4, treatment: *n* = 5, and untreated: *n* = 38), and an analysis including only adult males yielded qualitatively the same results.

The effect of the treatment on extra-pair siring success may be strongest in males that were exposed to the light treatment 1) on more mornings or 2) on mornings when more females in the local neighborhood were fertile. We, therefore, examined within the group of light-treated males whether the strength of the treatment was associated with extra-pair siring success. To this end, we determined for each day of the experimental period how many females 1) in a male’s first-order neighborhood and 2) in a male’s first- and second-order neighborhood were in their peak fertile period. We defined the “peak fertile period,” which is the period when females are most receptive to extra-pair fertilizations, as between 5 and 1 days before the female laid her first egg (see Schlicht et al. 2014a). For each male, we summed this number across all days on which they were manipulated. For example, a male that was exposed to the treatment on four mornings, during which three, five, six, and four females in the local neighborhood were fertile, respectively, received a score of 18. We used Thiessen polygons to estimate territory boundaries (Valcu and Kempenaers 2010) and considered a female to be a first-order neighbor of the focal male if the female’s territory shared a border with that of the roosting male, and a second-order neighbor if they were separated by one territory. We did not consider higher-order neighbors, because males rarely sire extra-pair offspring with such females (Schlicht et al. 2014b). We constructed a general linear model with a binomial error structure, with whether a male sired at least one extra-pair young (yes/no) as the response variable and with treatment score (the sum of the number of fertile females in the neighborhood; first-order neighbors only, or first- and second-order neighbors combined) and male age as fixed effects. We also ran the analysis including only adult males, which yielded qualitatively the same results. Adding the number of mornings on which a male was treated and the number of fertile neighboring females (fertile on at least one experimental morning) as two separate variables did not change the conclusions.

Finally, we examined whether emergence time predicted the probability of siring extra-pair young, separately for males in the experimental and in the control treatment. We used general linear models with a binomial error structure, with whether a male sired at least one extra pair young (yes/no) as the response variable and with emergence time (averaged across all days over the experimental period) and male age as fixed effects. We also ran the analyses including only adult males, which yielded qualitatively the same results.

## RESULTS

### Effects of the treatment on emergence time

Males exposed to the light treatment emerged from the roosting box significantly earlier (mean ± SD: 42 ± 11 min before sunrise, range: 66–19 min before sunrise) than males exposed to the control treatment (mean ± SD: 31 ± 6 min before sunrise, range: 41–5 min before sunrise; Table 1, Figure 1). Before the onset of the experiment, there was no difference in emergence time between males in the experimental and control group (Table 1, Figure 1).

**Table 1:** Results of linear mixed models examining the difference in emergence time (in minutes) between males in the experimental group (*N* = 31) and males in the control group (*N* = 15) before and during the experimental light treatment

		Estimate	SE	*t*	*P*
Before experiment	*(Intercept)*	−29.41	1.28		
	Treatment	−1.61	1.55	−1.03	0.31
During experiment	*(Intercept)*	−29.10	1.87		
	Treatment	−12.31	2.28	−5.40	<0.001

### Effect of the treatment on extra-pair siring success

**Figure 2:**
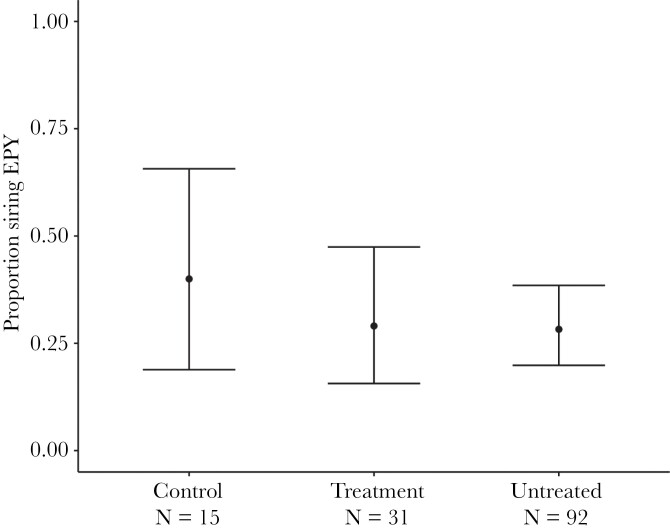
The proportion of males that sired at least one extra-pair young in the control treatment (*N* = 15), the experimental treatment (*N* = 31), and the untreated group (*N* = 92). Dots and error bars show model-predicted values and 95% confidence intervals.

Males in the experimental group did not differ significantly in the likelihood of siring extra-pair young (29%) from males in the control group (40%) or in the untreated group (28%) (Table 2, Figure 2). If anything, the difference was in the opposite direction as predicted, that is, experimental males were somewhat less likely to sire extra-pair offspring than males from the control group, but similar to the untreated group.

**Table 2:** Results of a generalized linear mixed model examining the effect of the experimental treatment and age (yearling versus older) on the likelihood of siring at least one extra-pair young. Values of the control group (*N* = 15) and the untreated group (*N* = 92) are relative to the experimental (light-treated) group (*N* = 31)

	Estimate	SE	*Z*	*P*
*(Intercept)*	−3.71	1.04		
Treatment (untreated)	0.28	0.48	0.59	0.55
Treatment (control)	0.65	0.68	0.95	0.34
Age (adult)	1.49	0.50	3.01	0.003

**Figure 3:**
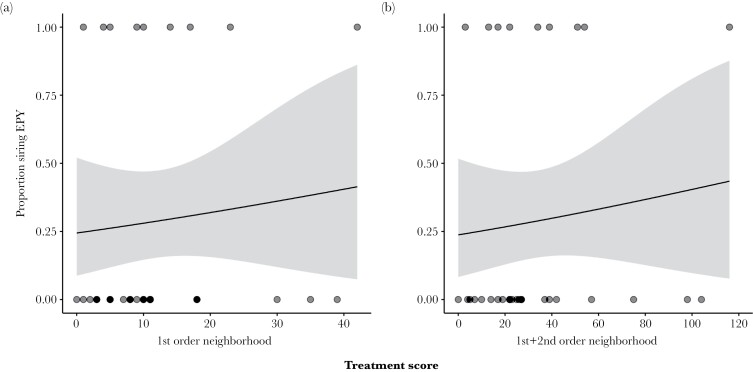
Relation between the likelihood of siring at least one extra-pair young and the strength of the manipulation within the light-treated group (*N* = 31). The treatment score reflects the number of mornings on which a male was exposed to the light treatment and the number of females in the local neighborhood that were fertile on those days (see methods). (a) Fertile females in the first-order neighborhood, (b) fertile females in the first- and second-order neighborhood combined. Lines and shaded areas show model predictions and 95% confidence intervals. Dots at the top and bottom show the treatments score of males that did and did not sire at least one extra-pair young, respectively.

Within the group of males exposed to the light treatment, there was no significant effect of the strength of the treatment on extra-pair siring success. Neither the summed number of fertile females within the first-order neighborhood nor the summed number of fertile females in the first- and second-order neighborhood combined predicted whether a male had sired extra-pair young (Table 3, Figure 3).

**Table 3:** Results of generalized linear mixed models examining the relationship between the strength of the experimental treatment (light-treated males only) and the likelihood of siring at least one extra-pair young (*N* = 31 males). Treatment score reflects the number of mornings on which a male was exposed to the light treatment and the number of females in the male’s local neighborhood (first order, or first + second order) that were fertile on the mornings that male was treated (see methods). Although age is a known predictor of extra-pair siring success, the number of yearling males was small (*N* = 5) and the effect, therefore, not statistically significant

		Estimate	SE	*Z*	*P*
First-order neighborhood	*(Intercept)*	−2.42	2.44		
	Treatment score	0.02	0.04	0.61	0.54
	Age (adult)	0.67	1.22	0.55	0.58
First + second-order neighborhood	*(Intercept)*	−2.47	2.45		
	Treatment score	0.01	0.01	0.67	0.50
	Age (adult)	0.68	1.22	0.56	0.58

### Effect of emergence time on extra-pair siring success

**Figure 4:**
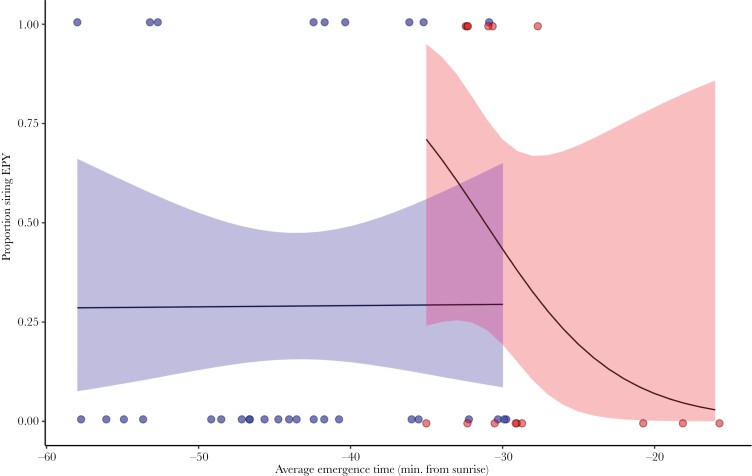
The relationship between emergence time (individual averages over the experimental period) and the probability of siring at least one extra-pair young for males in the experimental treatment (blue, *N* = 31) and in the control treatment (red, *N* = 15). Lines and shaded areas show model-predicted values and 95% confidence intervals. Dots at the top and bottom show the average emergence time of males that did and did not sire at least one extra-pair young, respectively.

Within the experimental group, male emergence time was not related to the likelihood of siring extra-pair young (Table 4, Figure 4a). Within the control group, males that emerged earlier were more likely to sire extra-pair young, but this effect was not statistically significant (Table 4, Figure 4b).

**Table 4:** Results of generalized linear mixed models examining the relationship between emergence time (average over the experimental period) and the probability of siring at least one extra-pair young, separately for males in the experimental (light-treated, *N* = 31) and control treatment (*N* = 15). Although age is a known predictor of extra-pair siring success, the number of yearling males was small (experimental group: *N* = 5, control group: *N* = 4) and the effect, therefore, not significant

		Estimate	SE	*Z*	*P*
Experimental males	*(Intercept)*	−2.01	3.24		
	Emergence time	<0.01	0.05	−0.02	0.98
	Age (adult)	0.58	1.20	0.48	0.63
Control males	*(Intercept)*	−52.41	9947.46		
	Emergence time	−0.37	0.26	−1.46	0.14
	Age (adult)	20.99	4973.73	<0.01	1.00

## DISCUSSION

We exposed blue tit males to artificial light in their roost about half an hour before their natural emergence time during the period when fertile females were present in the population. The light treatment significantly advanced their emergence time, such that almost all experimental males emerged before the control males. However, the treatment did not have any consequences for the males’ extra-pair siring success. Moreover, among the experimental males, there was no relationship between emergence time and the likelihood of siring extra-pair young. In contrast, in the control group, the predicted relation between emergence time and extra-pair siring success was observed, although it was not statistically significant. This suggests that emerging early may not affect extra-pair siring success, perhaps because females are not active yet at that time and unavailable for mating, or because experimental males did not show behaviors that are used by females as a signal of male quality. Our study thus challenges the notion that the timing of emergence per se is an important factor underlying variation in extra-pair siring success.

One possible explanation for why our treatment did not affect the extra-pair siring success of the treated males is that we were unable to manipulate males on every morning during the period when potential extra-pair mates were fertile. If many females were fertile on the mornings when a male was not manipulated, the treatment may not have been sufficient to have measurable effects on extra-pair siring success. However, even the males that received the strongest treatment, that is, those that were manipulated on more mornings and on mornings with more fertile females in their neighborhood, were not more likely to sire extra-pair young.

Our experimental results do not allow to conclude that early-morning behavior is unimportant for extra-pair siring success. Although males that were exposed to the light treatment emerged earlier from their roosting box, we did not track their behavior after they left the box. Thus, it is possible that these males did not pursue extra-pair mating earlier than they would normally, for instance, because they waited for light levels to increase. Furthermore, the timing of the start of the dawn chorus might be the trait that is causally linked to extra-pair siring success, and this may not have been affected by our treatment. Thus, while we successfully advanced emergence time, behaviors that are likely relevant for extra-pair mating success (e.g. singing, pursuing copulations) may not have been affected. Previous studies that exposed blue tits and other birds to artificial light showed that they did start singing earlier when exposed to light (Kempenaers et al. 2010; da Silva et al. 2014, 2016), suggesting that males that emerged earlier also started displaying earlier. However, in those studies, the entire local environment was exposed to strong lighting, whereas in our experiment only the roosting cavity itself was lit.

An alternative experimental approach to further address the importance of early-morning behavior is to delay (rather than advance) the emergence time of males. Such manipulation should result in lower extra-pair siring success if early emergence or behaviors that are expressed soon after emergence from the roost are causally related to extra-pair siring success. A simple approach could be to temporarily keep the nest-box hole closed in the early morning (see Kempenaers et al. 1998), but we caution that in the blue tit this led to nest abandonment. We previously delayed the emergence time of roosting females by presenting predator models in front of the breeding box (Santema et al. 2020) and a similar manipulation could be done for males. However, any observed effects on behavior after emergence could then also be due to the confrontation with a predator.

Despite more than two decades of intensive research on extra-pair paternity, still relatively little is known about the copulation behavior that underlies patterns of extra-pair paternity (Brouwer and Griffith 2019; Lifjeld et al. 2019). The main reason for this is that (extra-pair) copulations are typically brief and difficult to observe in free-living birds with traditional methods. Many studies, therefore, rely on indirect measures of extra-pair mating behavior, such as extra-pair siring success. Between-male variation in extra-pair siring success is likely linked to several factors and does not necessarily reflect variation in extra-pair mating activity (Brommer et al. 2007; Vedder 2022). Recent advances in tracking technology such as the development of miniaturized radio tags (e.g., Ripperger et al. 2020) may make detailed monitoring of interactions between males and (extra-pair) females feasible.

In summary, despite the well-documented relationship between early-morning activity and extra-pair siring success, male blue tits whose emergence time was experimentally advanced were not more likely to sire extra-pair young. This challenges the hypothesis that the timing of emergence and extra-pair success are causally related. While our results cannot conclusively show that behavior during the early morning is unimportant for extra-pair siring success, they at least suggest that it is premature to assign an important role to activity during this time of the day. Alternative experimental approaches and more detailed behavioral data are needed to advance our understanding of extra-pair mating behavior and resolve the importance of early morning activity. While much research has focused on the evolutionary causes and consequences of extra-pair paternity, the behavioral mechanisms underlying variation in extra-pair siring success remain poorly understood.

## Notes

We are grateful to Agnes Türk, Andrea Wittenzellner, Margherita Cragnolini, and all other people who contributed to the fieldwork for data collection, to Peter Loës and Peter Skripsky for the development and maintenance of the smart nestbox system, to Sylvia Kuhn and Alexander Girg for genotyping, to Mihai Valcu for managing the database, to Emmi Schlicht and Lotte Schlicht for discussion, and to Amanda Ridley and two anonymous reviewers for suggestions that helped improve the manuscript.
